# Editorial: Cold, heat and hypoxia as a medical tool: the use in a healthy and diseased population

**DOI:** 10.3389/fphys.2024.1380395

**Published:** 2024-02-14

**Authors:** Erich Hohenauer, Slavko Rogan, Ron Clijsen

**Affiliations:** ^1^ Department of Business Economics, Health, and Social Care, University of Applied Sciences and Arts of Southern Switzerland, Landquart, Switzerland; ^2^ Department of Physiotherapy, International University of Applied Sciences THIM, Landquart, Switzerland; ^3^ Department of Neurosciences and Movement Science, University of Fribourg, Fribourg, Switzerland; ^4^ Department of Health, Bern University of Applied Sciences, Bern, Switzerland

**Keywords:** hypoxia, cold, heat, health, therapy

## 1 Introduction

In the complex interplay between humanity and the environment, the profound effects of environmental stressors on human health unfold as an evolving narrative. In recent years, scientific exploration has increasingly focused on the potential advantages of intentional exposures to environmental stressors, such as cold, heat, and hypoxia (Brunt and Minson, 2021; Allan et al., 2022; Burtscher et al., 2023). The recognition of these stressors as effective tools for enhancing health and wellbeing might mark the onset of a new era in therapeutic possibilities.

The appeal lies in the concept of hormesis, where controlled exposure to mild stressors triggers adaptive responses within the body, leading to increased resilience and improved physiological function. Cold therapies, encompassing practices like ice baths and cryotherapy, hold the promise of anti-inflammatory effects and expedited physical recovery (Hohenauer et al., 2015; Garcia et al., 2021). Heat therapy, manifested through saunas and hot-water immersion, is acclaimed for its cardiovascular benefits and potential cognitive enhancements (Von Schulze et al., 2020; Brunt and Minson, 2021). Hypoxic training, mimicking conditions at high altitudes, has emerged as a strategy to bolster endurance and optimize oxygen utilization (Huang et al., 2023).

However, as we navigate the promising terrain of environmental stressors as therapeutic tools, we must also grapple with the intricacies of their physiological consequences. Exposure to extreme temperatures and diminished oxygen levels requires a nuanced understanding how to induce beneficial, stress-induced adaptations rather than potential harms.

This scientific article Research Topic aims to explore the two-sided aspects of environmental stressors, investigating their potentials to positively impact human health and the physiological complexities that determine the balance between intended therapeutic effects and unintended outcomes. This Research Topic presents current scientific findings, describing the effects of these stressors on various aspects of human physiology. In this endeavor, the current Research Topic provides evidence-based knowledge that accurately exploits the potential benefits of environmental stressors and recognizes the complex interplay between these stressors on the physiological system of our body.

## 2 Research Topic contributions

This compilation of research articles provides insights into diverse physiological phenomena, spanning from the impact of hypoxic exposure on asthmatics to the effects of cold-water immersion on cardiovascular health. Through investigations into high-altitude adaptability, glucose regulation during exercise, recovery strategies, and more, these studies contribute significantly to our understanding of human physiology across diverse conditions.

Saxer et al. explored acute and subacute effects of hypobaric hypoxic exposures on individuals with mild asthma, undergoing a 3-week rehabilitation program at 3,100 m. Pulmonary artery pressure increased with acute altitude exposure, accompanied by elevated pulmonary vascular resistance, heart rate, and decreased oxygen saturation. Extravascular lung water increased acutely but returned to baseline after 3 weeks. Tee et al. studied glucose regulation in overweight adults during low-intensity exercise under normoxia, moderate hypoxia, and high hypoxia. Post-exercise glucose and insulin responses were lower in moderate hypoxia, suggesting it as an effective stimulus for glucose regulation without excessive stress. Heart rate increased in high hypoxia, indicating physiological strain. Liu et al. identified urine biomarkers for high-altitude adaptability and stamina. High-stamina individuals showed elevated white blood cell counts and specific urine protein expressions. The study introduced potential biomarkers for screening individuals adapting to high altitudes with sustained endurance. Kagelman et al. investigated peripheral skin cooling as a countermeasure during hyper-gravity exposure. Peripheral skin cooling showed no significant impact on hemodynamics, suggesting potential limitations or insufficient cooling. Honorato et al. explored the impact of hypobaric hypoxia during flight on cardiac autonomic function. Hypoxia decreased heart rate variability, indicating sensitivity to hypoxic conditions. Wang and Hurr studied an analgesic cream’s effects during temperate-water immersion for exercise-induced hyperthermia. The cream enhanced cooling effects, demonstrated by increased cutaneous vascular conductance and core body heat loss, providing a cost-effective means for improved cooling during exercise-induced hyperthermia. Geng et al. investigated oxygen uptake and deoxyhemoglobin changes in athletes during incremental exhaustive exercise under various conditions. Hypoxia and high temperature decreased exercise capacity, suggesting potential factors contributing to peripheral fatigue under different environmental conditions. Jackman et al. explored acute physiological responses to hot-water immersion following resistance exercise. Hot-water immersion increased intramuscular temperature, suggesting its viability for heat therapy, but did not significantly impact muscle function or soreness. Treigyte et al. examined the effects of cold-water immersions on muscle force and contractility during and after electrically induced contractions. Intermittent/prolonged immersions induced a less pronounced contractile transition, potentially due to reduced vasoconstriction response and enhanced blood perfusion during immersion. Versteeg et al. investigated the effects of a 3-week repeated cold-water immersion on leukocyte counts and cardiovascular factors in healthy men. While leukocyte counts decreased in both cold-water immersion and the control group, cardiovascular factors showed reductions only in the cold-water group.

## 3 Perspective

In summary, these studies provide insights into the physiological responses under various conditions, offering valuable information for optimizing interventions and countermeasures in situations such as high-altitude exposure, exercise-induced hyperthermia, and hypoxic environments, leading to promising paths for future research.

Based on the findings from Saxer et al. further investigations into hypobaric hypoxic exposures into sustained adaptability in individuals with mild asthma are needed. Tee et al.’s findings on glucose regulation under varying hypoxic conditions invite exploration into molecular mechanisms, particularly the benefits of moderate hypoxia for metabolic health. Liu et al.’s identification of urine biomarkers introduces avenues for personalized screening in high-altitude adaptation. In the study from Kagelmann et al., the exploration of peripheral skin cooling during hyper-gravity exposure highlights the need for optimal cooling strategies. Honorato et al. suggests monitoring heart rate variability in future research for enhanced hypoxia detection during flights. Wang and Hurr’s insights into an analgesic cream’s efficacy in exercise-induced hyperthermia present cost-effective cooling options. The need for future investigations regarding the molecular mechanisms that underlie the counteractive vasodilatory effect of analgesic creams was highlighted in this study. The study results from Geng et al. regarding oxygen uptake calls for further exploration into peripheral fatigue factors under diverse environmental conditions. Jackman et al.’s exploration of hot-water immersion for heat therapy prompts considerations of its long-term impact on muscle function. Treigyte et al.’s study on cold-water immersions hints at the potential role of sex differences that should be considered in future research. In the study from Versteeg et al., future studies should incorporate larger sample sizes to investigate the true effects of repeated cold-water immersions on immune and cardiovascular factors.

In summary, the included studies in this Research Topic significantly advance our understanding of the effects of cold, heat, and hypoxic exposures. The investigations provide valuable insights into various aspects of the interventions, demonstrating the potential benefits and mechanisms. In conclusion, while these studies have greatly enhanced our physiological knowledge, they also emphasize the ongoing need for additional research in this area to unravel the physiological impact of these environmental stressors to reach definite outcomes.
